# LTR retrotransposons reveal recent extensive inter-subspecies nonreciprocal recombination in Asian cultivated rice

**DOI:** 10.1186/1471-2164-9-565

**Published:** 2008-11-27

**Authors:** Hao Wang, Zhao Xu, Hongjie Yu

**Affiliations:** 1 T-life Research Center, Department of Physics, Fudan University, Shanghai 200433, PR China; 2 College of Life Science, Zhejiang University, Hangzhou 310008, PR China

## Abstract

**Background:**

Long Terminal Repeats retrotransposons (LTR elements) are ubiquitous Eukaryotic transposable elements (TEs). They are considered to be one of the major forces underlying plant genome evolution. Because of relatively high evolutionary speed, active transposition of LTR elements in the host genomes provides rich information on their short-term history. As more and more genomes, especially those of closely related organisms, have been sequenced, it is possible to perform global comparative study of their LTR retrotransposons to reveal events in the history.

**Results:**

The present research is designed to investigate important evolutionary events in the origin of Asian cultivated rice through the comparison of LTR elements. We have developed LTR_INSERT, a new method for LTR elements discovery in two closely related genomes. Our method has a distinctive feature that it is capable of judging whether an insertion occurs prior or posterior to the divergence of genomes. LTR_INSERT identifies 993 full-length LTR elements, annotates 15916 copies related with them, and discovers at least 16 novel LTR families in the whole-genome comparative map of two cultivated rice subspecies. From the full-length LTR elements, we estimate that a significant proportion of the rice genome has experienced inter-subspecies nonreciprocal recombination (ISNR) in as recent as 53,000 years. Large-scale samplings further support that more than 15% of the rice genome has been involved in such recombination. In addition, LTR elements confirm that the genome of *O. sativa ssp. indica *and that of *japonica *diverged about 600,000 years ago.

**Conclusion:**

A new LTR retrotransposon identification method integrating both comparative genomics and *ab initio *algorithm is introduced and applied to Asian cultivated rice genomes. At whole-genome level, this work confirms that recent ISNR is an important factor that molds modern cultivated rice genome.

## Background

The origin (domestication) of Asian cultivated rice has intrigued the science community for decades and is still hotly debated. At DNA level, origin means the beginning of the sequences of agricultural important loci. It is well established that besides vertical transmission of genetic material from parent to offspring, horizontal process that genetic information move across mating barriers plays important role in the genome evolution [1]. In rice species, it is widely accepted that horizontal process such as gene flow and introgression have occurred among *O. sativa*, *O. nivara *and *O. rufipogon*; and many studies of population genetics, e.g. see reviews in [2,3], have explored horizontal process between cultivated and wild rice. However, few researches have focused on globally investigating, from the angle of genome comparison, the horizontal process between *indica *and *japonica*, the two reproductively isolated subspecies of *O. sativa*. The difficulties come from at least two aspects: firstly, although horizontal process makes target sites showing higher conservation than it should be, observed high conservation does not necessarily the result of horizontal process because low substitution rate will also lead to that, as in the case of ultraconserved sequences in animals [4,5]. Secondly, two subspecies have relatively short history, thus the statistical analysis of slowly evolving sites such as genes may brings biased results because of too small number of substitutions. Therefore it is important to find proper material to perform the investigation.

TEs are mobile repetitive DNA that have been found in all eukaryotic genomes investigated so far [6-9]. LTR retrotransposons are class I TE that transpose in a "copy and paste" mode via intermediate RNA. The typical structural characters of an LTR retrotransposon include: (1) two highly similar LTR sequences; (2) 4–6 bp target site repeats (TSR) at 5' and 3' ends; (3) primer binding site (PBS) downstream of 5' LTR and polypurine tract (PPT) upstream of 3' LTR; (4) protein domains important for transposition [10]. They are predominant components of large plant genomes and their amplification and deletion have been taken as one of the major forces underlying the remarkable variation of plant genome size [11-14].

Historical information in full-length LTR elements allows one to trace the birth date and substitutions in elements. If a LTR element inserts in a non-conserved region prior to the divergence of two genomes but its descendants show significant higher conservation than expected, the region must be involved in recently recombination that made alleles in two genome identical (see below). In addition, because of relatively high evolutionary speed, the amplification of LTR retrotransposons in short period makes them ideal markers to study short-term evolution. With the two features, LTR elements can be used to test recent horizontal process between closely related genomes.

This report is designed to explore important events in the evolution of Asian cultivated rice (*Oryza sativa L*.) from the angle of whole-genome comparison of LTR retrotransposons, with emphasis on revealing the ISNR between *indica *and *japonica*. We have developed LTR_INSERT to discover LTR retrotransposons in two related genomes. Unlike other methods, besides reporting the structure and location, LTR_INSERT tells whether an element inserts prior or posterior to the divergence of two genomes. By applying LTR_INSERT to *indica *and *japonica *genome [15,16], we identified 993 full-length LTR elements, annotated 15916 copies related with them, and discovered at least 16 novel LTR families. We found that many of these elements had significantly higher degree of sequence conservation than expected and that such high conservation was caused by ISNR. The subsequent large-scale sampling of protein-coding genes and random genomic sites showed that the phenomenon was not restricted to LTR retrotransposons and at least 15% of the genome was involved in ISNR in the recent past. In addition, LTR elements provided two independent evidences to confirm that two genomes diverged about 600,000 years ago.

## Results and discussion

### Overview of LTR retrotransposons identified by LTR_INSERT

**Figure 1:**
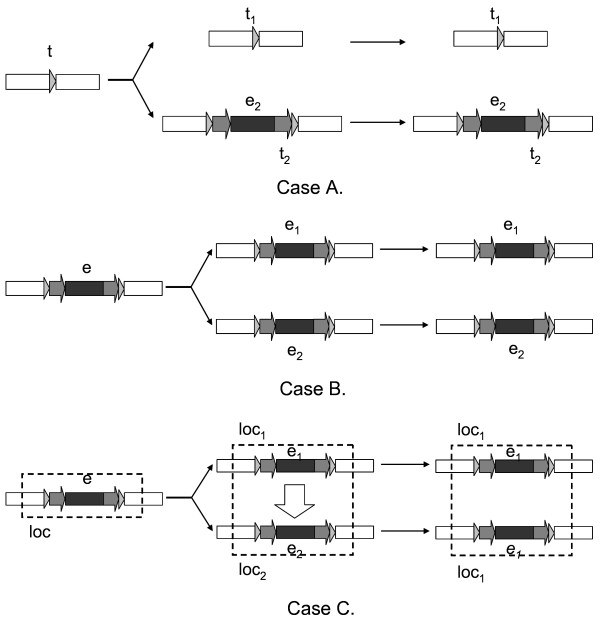
**The origin of allelic specific and shared LTR retrotransposons**. Case A: The origin of a specific element. Site *t *(grey triangles) in the common ancestor corresponds *t*_1 _and *t*_2 _in the two descendants. After two genomes diverge, an specific element *e*_2 _inserts at *t*_2_. This pair of alleles shows well-matched flanking sequences (white regions) with *e*_2 _being the alignment indel. Case B: The origin of a pair of shared elements. *e *inserts into the common ancestor and has *e*_1 _and *e*_2 _as its orthologous descendants. Case C: ISNR between a pair of shared elements. In recombination, *loc*_2 _is converted by *loc*_1 _and the sequence of *loc*_2 _become identical with that of *loc*_1_. Specifically, *e*_2 _is converted by *e*_1_.

A pair of allelic shared LTR retrotransposons is two highly similar elements that are found at the same locus in two closely related genomes. In contrast, if an element is present in one genome but its counterpart absent in the other, it is called an allelic specific element (Figure 1). Usually, allelic shared LTR retrotransposons derive from insertions prior to the divergence of two genomes (pre-divergence insertions), while specific ones though insertions posterior to that (post-divergence insertions). For simplicity, we use specific and shared to replace allelic specific and allelic shared sometimes in the following description.

LTR_INSERT scanned the *indica-japonica *comparative map ([see Additional file 1] and Methods) and identified 993 full-length elements, including 246 (i.e. 123 pairs) shared and 747 specific ones. Among them, 601 specific elements were discovered in *japonica *and 146 in *indicia*. LTR_INSERT also identified 715 allelic specific Solo-LTRs (392 in *japonica *and 323 in *indica*) related with them. With well-aligned flanking sequences and TSRs, most of the solo-LTRs must derive from intra-element unequal recombination of specific full-length elements. They also represent post-divergence insertions because recombination between different elements usually results in solo-LTR without TSR and well-aligned flanking sequences [17]. The subsequent BLAST search (E-value: *e*^-10^) retrieved more than 88,000 potential LTR copies related with the full-length elements. Discarding short ones ([see Additional file 2], section 1), we obtained a total of 15,916 LTR copies in the two genomes. In them, we extracted 3,102 pairs of shared copies (including full-length and truncated ones) that did not overlap with annotated gene models. Representing LTR-related intergenic alleles in two subspecies, these copies were used to test ISNR between two genomes (see section "Whole-genome samplings reveal that at least 15% of rice genome has experienced ISNR" below).

Because the description of LTR elements themselves is not the main purpose of this report, we put in appendix ([see Additional file 2]) the detailed analysis of the rice LTR families, including the criteria of family, the phylogenetic analysis and the dynamics of elements. Here, we only give a brief account of major results. We identified 195 rice LTR families (Table S2–4) with 80 being reported for the first time (Table S2–3). 16 of the 80 novel families (Table S2) had no hit with any known elements in GenBank [18], TIGR Plant Repeat Database [19], Repbase [20] and RetrOryza [21]. Although most of the 16 families lacked reliable long ORFs similar to know domains, some of them were quite active. We detected the RT domains in 135 families and the phylogenetic analysis based on the domains supported previous classification of rice LTR retrotransposons into Copia and Gypsy superfamilies (Figure S2). The investigation of the amplification pattern of rice

LTR families in time and spacial dimensions reveals several features ([see Additional file 2], section 3): (1) LTR retrotransposons have been active since the divergence of two genomes. (2) Although there was five-fold more full-length specific elements identified in *japonica *than in *indica*, the analysis of all the LTR copies showed that their abundance was relative balance between two lineages. (3) 80% of the post-divergence insertions were driven by 20% of highly active families (Table S6). These predominant families had been active in the common ancestor and the divergence event did not significantly change their activity. (4) The distribution of LTR elements (Figure S3) was non-random across the rice genome: the LTR density tends to decrease from centromeric regions to 5' and 3' ends of chromosomes. Besides centromeric neighborhoods, 5' ends of chromosomes were also LTR-dense regions.

### LTR retrotransposons reveal recent extensive inter-(sub)species nonreciprocal recombination in cultivated rice

#### History inside LTR retrotransposons and ISNR

Full length LTR elements store information of their transposition history in the LTRs [22,23]. When transposing, two identical LTRs synthesize from the same template, then they diverge with nucleotide mutations cumulating as time elapses. Given the average rate of nucleotide substitution (denoted as *r*) and sequence distance (denoted as *d*), insertion date (denoted as *T*) can be estimated by *T *= *d*/2*r*. In this study, we use *r *= 1.3 × 10^-8^/*site*/*yr*, as suggested in [24]. Further more, the comparison of specific and shared LTR elements in related species provides more information.

Specific elements are mainly generated through post-divergence transposition events (Figure 1, Case A). Ideally, the distance between their two LTRs (denoted as *d*_*intra*_) should be less than the distance corresponding to the divergence time (denoted as *D*). Besides post-divergence insertion, the accurate deletion of a shared element in one genome (intra-element reversible recombination) also makes its counterpart a specific element. However, the probability is rather low because this requires that recombination occurs at the 4–6 bp TSRs. In fact, just like insertions of LINEs and SINEs, transposition of LTR retrotransposons is almost irreversible. Even though such recombination occurs, it can be recognized by the age of this element: its *d*_*intra *_value is greater than *D *now. In short, most specific elements represent post-divergence transposition events.

Sources of shared elements can also be traced by analyzing history information inside full-length elements. When two elements are orthologs, the distance between them (denoted as *d*_*inter*_) is just *D *and because the insertion occurs prior to the divergence, *d*_*intra *_> *D *holds. Overall one has *d*_*inter *_= *D *<*d*_*intra *_for orthologous shared elements (Figure 1, Case B). Indeed, when no ISNR occurs, this is almost the unique source of shared elements because the probability that two independent transposition events in two species just insert into the same locus is low, the rather that they are highly similar.

Shared elements may be converted by ISNR and are not orthologs any more (Figure 1, Case C). In this process, an allele in one genome is converted by its counterpart in the other through nonreciprocal homologous recombination. We use the words "nonreciprocal homologous recombination" according to the suggestion in Li (1997) [25], to denote a type of recombination that "one sequence is changed whereas the other is not". When ISNR occurs, two alleles become identical and the history before ISNR is removed. As a result one observes that their pairwise distance is less than *D*. Specifically, if the two alleles cover a pair of shared elements, one expect to see *d*_*inter *_<*D *<*d*_*intra *_because ISNR, as a type of recombination, does not destroy the structure of elements and *d*_*intra *_still reflects the date of insertion, which occurs in the common ancestor. LTR retrotransposons usually have relative high evolutionary speed, so a pair of elements is highly probable converted by ISNR if its *d*_*inter *_value is significantly less than *D*.

**Figure 2:**
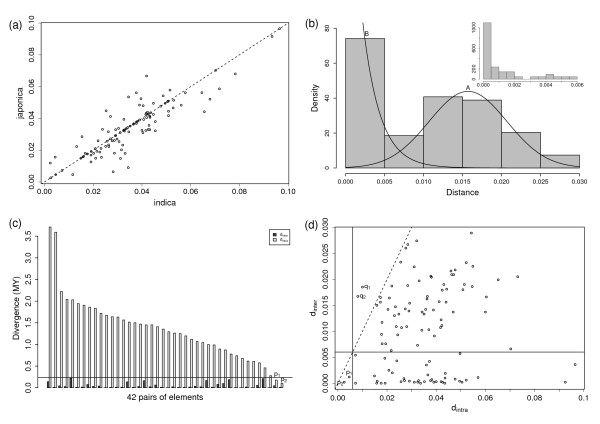
**Inter- and intra- element distance of shared full-length elements**. (a) Scatterplot of *d*_*intra *_of the 108 shared pairs. (b) The distribution of *d*_*inter *_of the shared pairs. Most of the Group-A elements are orthologs and Peak-A is located at 0.0157 (0.6 MYA). Two distributions separate at 0.006 (0.23 MYA). The insert figure gives that *d*_*inter *_≤ 0.0014 (53,000 years B.P.) holds in 74% of the Group-B elements,. (c) *d*_*inter *_and *d*_*intra *_of the Group-B elements. Each pair of bars represent *d*_*inter *_(grey column) and *d*_*intra *_(white column) values of a pair of elements. From left to right, pairs are sorted by their *d*_*intra *_values. (d) Scatterplot of *d*_*inter *_versus *d*_*intra *_of the shared pairs. The two solid lines give *d *= 0.006 in X- and Y- axis. The dashed line is *d*_*inter *_= *d*_*intra*_.

Theoretically, there are other possible ways to generate shared elements, e.g. at a loci where one allele (say allele x) contains a specific element but its counterpart (allele x') dose not, recombination converts x' by x. In this case, the shared pair shows *d*_*inter *_<*d*_*intra *_<*D*. We found that two pairs of elements in rice (*p*_1 _and *p*_2 _in Figure 2(c) and 2(d), see below) had such time pattern. Although small in sample size, the two examples indicate that such type of recombination might occur in the past.

In short, most shared and specific elements reflect pre- and post- divergence transposition events, respectively. The relationships among *d*_*inter*_, *D *and *d*_*intra *_give evolutionary events that generate and modify elements. We summarized the above discussion in Table 1. In practice, we infer that one of the relations hold and corresponding event took place in a group of elements based on high statistical significance.

**Table 1:** Source of specific and shared LTR retrotransposons

Type of element	Relationship of distances	Events in the evolution
Specific	*d*_*intra *_<*D*	Post-divergence insertion
	
	*d*_*intra *_> *D*	Pre-divergence insertion, then intra-element reversible recombination

Shared	*d*_*inter *_= *D *<*d*_*intra*_	Pre-divergence insertion
	
	*d*_*inter *_<*D *<*d*_*intra*_	Pre-divergence insertion, then ISNR between two genomes
	
	*d*_*inter *_<*d*_*intra *_<*D*	Post-divergence insertion. Recombination between two genomes generates one counterpart
	
	Other patterns	Insertion of two elements at the same locus, etc.

### Many LTR retrotransposon-contained loci have been converted by ISNR

We discarded elements of which LTRs were shorter than 200 bp and selected 825 (108 shared pairs and 717 specific ones) full-length elements for further analyses.

Each pair of elements has two *d*_*intra *_values, one from the *indica *member, the other from its *japonica *counterpart. As can be seen from Figure 2(a), the two values are overall equal or close to each other in pairs. Statistical analysis also supports this observation ([see Additional file 3] for details). Therefore, in the following discussion, we use the average of two values to represent *d*_*intra *_of each pair.

Figure 2(b) shows the distribution of the *d*_*inter *_values of the 108 shared pairs. This distribution is the superposition of an unimodal distribution A and a pulse-like distribution B. For a single splitting event and constant evolutionary rate, *d*_*inter *_of orthologous alleles should be close to a normal distribution with the mean value corresponding to the divergence date. When evolutionary rates are varied among loci, a flatter peak may appear in the distribution. On the other hand, when ISNR occurs with high frequency in a short period, a sharp increase should be observed at that time. Therefore the bimodal pattern here may be resulted from genetic material transfer on vertical and horizontal directions, respectively. This assumption is further supported by the following facts.

Since distribution A fitted the normal distribution *N*(*μ *= 0.0157, *σ *= 0.005) well (P-value = 0.98 in Kolmogorov-Smirnov test and 0.52 in Shapiro-wilk test), we identified elements belong to it (called Group-A elements) by selecting pairs of which *d*_*inter *_values were greater than 0.0059 (the value *μ *– 1.96*σ*). A total of 66 pairs were classified as Group-A elements and the left 42 pairs as Group-B elements (Figure 2(d)).

Unlike some previous researches to estimate divergence date based on *d*_*intra *_[24,26], we directly estimate the divergence time from *d*_*inter *_of orthologous elements: firstly, *D *= *d*_*inter *_holds in them because of normality of distribution A. Secondly, we find that *d*_*inter *_<*d*_*intra *_in almost all pairs. *q*_1 _and *q*_2 _in Figure 2(d) are two execptions, but they can be explained by random fluctuation (see below). These observations support that Group-A pairs are orthologs and two genomes diverged about 0.6 MYA (the date corresponding to *μ *= 0.0157).

Distribution B is a sharp spike at *d*_*inter *_= (0, 0.006) and the following characters are found in corresponding elements: (1) All of them show *d*_*inter *_<*d*_*intra*_. (2) *d*_*inter *_values of the 42 pairs are significantly (level of significance: 0.025) less than *D*. (3) Most of them (88%) have *d*_*intra *_values greater than *D *and 2/3 greater than 1 MY, the date corresponding to *μ *+ 2*σ *(Figure 2(c)). In summary, the inequality *d*_*inter *_<*D *<*d*_*intra *_holds in most of them. Further more, as can be seen from the insert figure in Figure 2(b), the great majority (74%) of Group-B pairs have *d*_*inter *_values less than 0.0014 (53,000 years B.P.), coincident with the time of the earliest evidence of modern human activities in South and East Asia, which are thought to be potential regions of rice domestication.

Most Group-B elements are not located in conserved genomic regions because they have great *d*_*intra *_values, which reflect that great number of mutations cumulated in these regions. Therefore it is hard to explain their small *d*_*inter *_values by conservation of loci. Because the above analyses have shown that the majority of Group-B elements meet the features of ISNR, we propose that they were involved in such process.

Providing more information than frequency histogram, scatterplot allows us to see more things. Figure 2(d) shows *d*_*inter *_versus *d*_*intra *_of the 108 pairs of shared elements. According to the above discussions, the solid horizontal line (*d*_*inter *_= 0.0059) separates pairs into Group-A (points above the line) and Group-B (below the line). *q*_1 _and *q*_2 _are two pairs that lie above the *d*_*inter *_= *d*_*intra *_line (dashed line). That is, their *d*_*inter *_> *d*_*intra*_. Since their *d*_*intra *_values are small, *d*_*inter *_are close to *μ *and the phenomenon is only observed in two pairs, it is reasonable to explain the 3% outliers by random fluctuation. *p*_1 _and *p*_2 _are two pairs located on the left of the vertical solid line, which means they have *D *> *d*_*intra *_significantly.

They also have *d*_*intra *_> *d*_*inter *_since they are located below the dashed line, but this relationship is not statistically significant because the sample size is rather low. Therefore, *p*_1 _and *p*_2 _might derive from inter-genome recombination of post-divergence insertions (Table 1), but the current work lacks enough samples to confirm this beyond doubt.

### Whole-genome samplings reveal that at least 15% of rice genome has experienced ISNR

**Figure 3:**
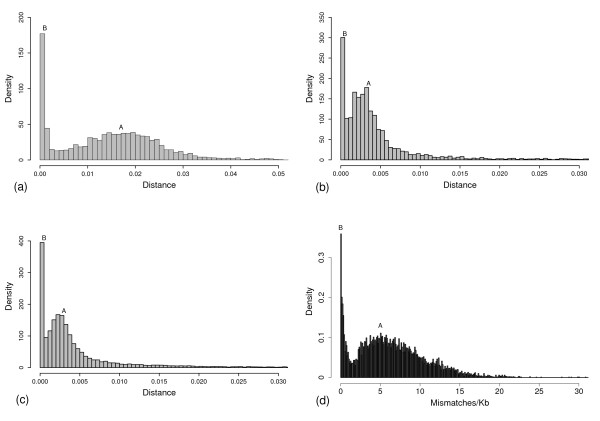
**The distribution of inter-subspecies distance in large-scale samplings**. (a) *d*_*inter *_of 3,102 pairs of LTR-related intergenic sequences. (b) *d*_*inter *_of the intron regions of 5,502 cDNA verified genes. Comparing to LTR-related loci, the average substitution rate in genic regions is 5–6 times slower. (c) *d*_*inter *_of the intron regions of 19,775 BGF predicted gene models. (d) Mismatch densities of 400,000 random samplings in the comparative map. The result gives that 15.3–18.7% of genomic sequences belong to Group-B.

Large-scale sampling further confirmed that the same bimodal pattern of *d*_*inter *_existed in the entire genome, including non-LTR-related regions. *d*_*inter *_is now defined as the distance between any pair of alleles and no longer restricted in shared LTR elements. Firstly, we investigated the 3,102 intergenic LTR regions (see section "Overview of LTR retrotransposons identified by LTR_INSERT"). Comparing to the shared full-length elements, the sample capacity increased 30 folds. The distribution and percentage of group-B elements (Figure 3(a), Table 2) are clearly consistent with Figure 2(b): a peak is located at 0.015–0.018 and the two distributions separate at 0.005–0.006. Next we calculated pairwise distance of intron regions of 5,502 pairs of cDNA verified genes and 19,775 pairs of BGF annotated gene models respectively (see Methods) and found the bimodal pattern as well (Figure 3(b) and 3(c)): the peak of distribution A is located at 0.003–0.004 and two distributions separate at 0.001–0.0015. The investigation of coding regions gave similar results ([see Additional file 4]).

**Table 2:** Proportion of Group-B sites

Type	Sample capacity	Percentage	
		Number (%)	Size (%)
FL LTR copy	108	25.9	31.2
LTR copy	3,102	27.9	31.7
Gene I^*a*^	5,502	26	17.3
Gene II^*b*^	19,775	24.5	19.9
Random Sampling	400,000	15.3 ~18.7^*c*^	15.3 ~18.7

^*a *^cDNA verified genes.

^*b *^BGF predicted genes.

^*c *^Different values result from varied window sizes.

Genes and LTR elements may not accurately tell the boundaries of ISNR loci because they are only subregions of the ISNR loci (Figure 1, Case C). We estimated the percentage of genomic sequences involved in ISNR by random sampling of sites in the comparative map: windows of a certain length are randomly placed in the map and for each window, the mean mismatches/Kb value (mismatch density in window) is calculated. Not preferring certain loci, the sampling provided less biased estimation of the percentage. The calculation of mismatch density in window of 5–10 Kb in 400,000 random samplings confirmed the bimodal distribution: distribution A and B separate at 1.5–1.8 mismatches/Kb and about 15.3%–18.7% of genomic sequences belong to Group-B (Figure 3(d) and Table 2).

**Figure 4:**
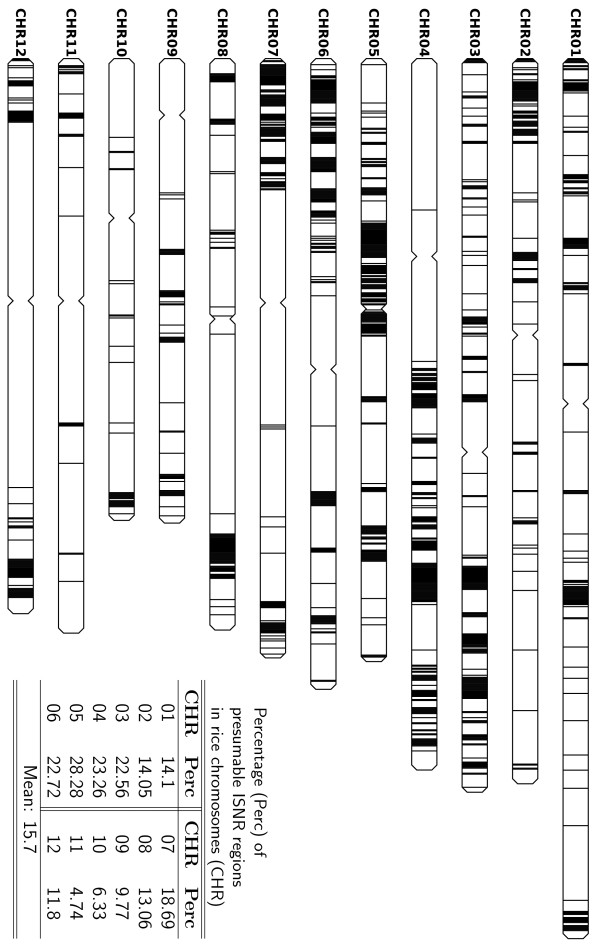
**Distribution of presumable ISNR regions in the rice genome**. This diagram is based on the *japonica *genome. The sliding window of 5 Kb shifts 1 bp in each step. Regions in which the average mismatch density ≤ 1.5 = *Kb *are drawn. Each line represents 50 Kb regions. A line is drawn only when there are at least 10 Kb sequences show ≥ 99.85% conservation. Therefore, many highly conserved "islands" shorter than 10 Kb are not shown here.

By using a sliding window scanning the comparative map, we obtained the distribution of presumable ISNR regions (regions that average mismatch density ≤ 1.5/*Kb*) in the rice genome. As can been seen from Figure 4, instead of evenly distributed along the twelve pseudochromosomes, ISNR regions are found to be more dense in chromosome 3–7, while sparse in chromosome 9–11. Our investigation has shown that the rice LTR elements tend to be more dence at the centromeric regions in all chromosomes (Figure S3). By contrast, ISNR regions do not occur in the centromeric regions of eleven chromosomes except chromosome 5. These results are consistent with that centromeric regions are rich in retroelements but contain regions with suppressed recombination. Although ISNR regions seem to appear in the centromeric region of chromosome 5, it should be caution that these highly conserved regions may actually not be in but near to that region because the centromeric regions are hard to sequence and we only obtained the approximate location information of centromeres from TIGR Rice Genome Annotation [19].

In summary, the same bimodal pattern of the distribution of *d*_*inter *_appears in all cases investigated, including full-length LTR elements, genes, intergenic and random genomic regions with the sample capacity ranging from one hundred to tens of thousands. The consistency indicates that a significant portion of rice genomes have experienced ISNR. The percentage is more than 15% of the rice genome and ISNR took place with high frequency in recent past. As the time is overlapping with the rising of modern human, it is highly probable that ISNR is related with the process of domestication.

It is important to emphasize that ISNR may take place directly between two subspecies or via other related genomes, e.g. those of wild rice. As a type of horizontal process, ISNR provides a mechanism to spread alleles among cultivated rice and their wild relatives. The present research confirms that it is an important force in the evolution of rice. Because of more than 20% protein-coding genes are belong to distribution B (Figure 3(b) and 3(c), Table 2), ISNR might have greatly influenced the domestication of important traits. Besides evidences of this study, a recent population genetics analysis has shown that *sh4*, the grain shattering gene, is once origin and spread in all cultivated rice via horizontal gene flow [27]. These results further indicate that ISNR, as well as other horizontal forces, may have had impact on the domestication important traits.

### Two genomes diverged about 600,000 years ago

**Figure 5:**
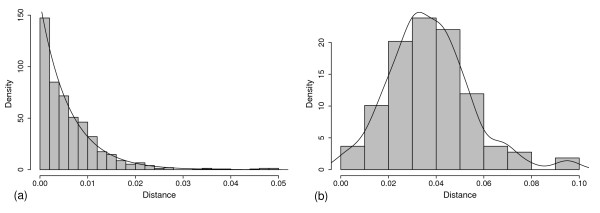
**Distribution of *d*_*intra *_of full-length elements**. (a) *d*_*intra *_of the 717 specific full-length elements. In more than 90% of them, the ages are less than 0.014 (0.54 MY). (b) *d*_*intra *_of the 108 pairs of shared full-length elements. In 90% of them, the ages are greater than 0.017 (0.65 MY). That two distributions separate at *d*_*intra *_= (0.014, 0.017) indicates that the two genomes diverged between 0.54 and 0.65 MYA.

The above normality and orthology analysis of *d*_*inter *_for the Group-A elements gave that peak-A reflected the divergence of two genomes and it occurred about 0.6 MYA. This argument can be further supported by *d*_*intra *_of elements. Because most of the specific elements insert posterior to the divergence of two subspecies and most shared ones insert prior to that, so the distributions of *d*_*intra *_of specific and shared elements give the upper and lower limit of the divergence date, respectively. As shown in Figure 5(a) and 5(b), the insertion dates of specific and shared elements naturally belonged to two periods. *d*_*intra *_values are less than 0.014 in 90% of specific elements while greater than 0.017 in the same percentage of shared pairs. This result well supports that most specific and shared elements represent post- and pre- divergence insertions, respectively. Since *d*_*intra *_= (0.014, 0.017) corresponds to 0.54–0.65 MYA, The fact that peak-A is located in the period is consistent with that distribution A reflects orthologous loci and two genomes diverged about 0.6 MYA, a greater value than some recent estimations [24,26,28-30].

### Evolutionary speed is 5–6 folds faster in LTR regions than in genes

Analogously, the distribution A of other cases should also represent distribution of orthologous loci. By comparing Figure 3(b) and 3(c) to Figure 2(c) and 3(a), it is easy to observe that the distance values corresponding to peak-A, peak-B, and the separation of two distribution are systematically 5–6 times less in gene regions. We also find that mean *d*_*inter *_value of introns and *d*_*s *_value of coding sequences have no significant difference in Group-A sequences ([see Additional file 4]). These results indicate that mean speed of substitution in LTR-related loci is 5–6 times faster than in genic regions and the value is higher than the estimation of [24].

### Extensive ISNR in rice add puzzles to the origin of cultivated rice

Traditionally, there are two models about the domestication of rice: the "single origin" hypothesis claims that the cultivated rice was only domesticated once and the differentiation of two subspecies was latter than the domestication of important traits, which took place no earlier than the beginning of agriculture [31-34]. On the other hand, the multiple origin" hypothesis argues that domestication occurred at least twice; two subspecies were cultivated from diverged wild populations and their progenitors had already diverged by the beginning of agriculture [35,36]. To date, the divergence time (or evolutionary distance) of the two rice genomes has been widely used as the decisive criterion for this question. Recently, researchers have tried to estimate the distance from varied loci and the "multiple origin" model seems to be supported by different molecular materials [26,29,37]. However, this criterion is valid only when the influence of horizontal process is trivial. If horizontal process was important in the early stage of domestication, as has been shown in this report, *indica *and *japonica *may have ancient diverged genomic "background" yet the domestication of important traits only took place once. In other words, traits might be domesticated first in one type of rice and the other subspecies originated from some far-related wild rice which hybridized with it and acquired these traits through ISNR. Besides this picture, ISNR can provide multiple possibilities on the rice domestication. For instance, groups of traits might be independently domesticated with host genomes and subsequently combined through ISNR to generate two types of cultivated rice [27].

This work reveals that rice genomes are mosaic, which reconciles the once-multiple origination conflict but complicates the origin of rice: one must face distinctive histories of traits now! To reconstruct the history of cultivated rice, ISNR, as well as other horizontal process, must be considered. Only when both vertical and horizontal forces are well understood, the domestication can be understood in-depth.

## Conclusion

We have introduced a new method, LTR_INSERT, to detect LTR retrotransposons in two related genomes and use them to explore the history of Asian cultivated rice. By discriminating between pre- and post-divergence transposition events, LTR_INSERT provides rich information on the evolution of both rice and LTR elements themselves. Through LTR elements and whole-genome scale loci samplings, we find that at least 15% of the rice genome has experienced ISNR in very recent past. In addition, the analyses of LTR elements also confirm that the two rice genomes diverged about 600,000 years B.P.. These results clearly show that ISNR has actively participated in shaping of the cultivated rice genome.

## Methods

### Genomic sequences and databases

Genomic sequences of *indica*, version 4 pseudochromosomes of *japonica *and Full-length cDNAs were downloaded from NCBI [18], TIGR [19] and KOME [38], respectively.

### Mining LTR retrotransposons in related genomes

We identified LTR retrotransposons through three main steps: (1) Construction of a whole-genome comparative map; (2) identification of specific and shared full-length elements by LTR_INSERT; (3) identification of other homologous copies including truncated ones through similarity search.

#### Construction of comparative map for indica and japonica

The comparative map was constructed by marker building and syntenic block alignment. *indica *genome was first fragmented by sequencing gaps, then all longer than 5 Kb segments were mapped to *japonica *with MUMmer [39] and highly convserved (similarity > 90%) homologous pairs were selected. These pairs, called long syntenic alleles (LSAs), established reliable correspondence between two genomes. Subsequently, a denser marker set was constructed by adding allelic cDNA verified genes to spacer regions of the LSAs: after eliminating redundant and short (size < 1 Kb) entries, KOME cDNAs were mapped to *japonica *pseudochromosomes, then their *indica *counterparts were identified by BLAT [40]. With these markers, the two genomes were partitioned into syntenic blocks. At last, each syntenic block was globally aligned and all the alignments were concatenated to accomplish the map. The detailed description of the indica-japonica comparative map is in Additional file 1.

#### LTR_INSERT and full-length LTR retrotransposon identification

LTR_INSERT is a new method to discover LTR retrotransposons in closely related genomes. To date, besides homology-search methods, some *ab initio *LTR retrotransposon finders have been developed [41-43]. Recognizing structural characters of elements, *ab initio *methods are able to discover novel elements yet may bring high false positive. Recently, Caspi and Pachter [44] have introduced comparative genomics to find TEs in fruit fly genomes. Their method searches out possible insertion events in related genomes and thus provides evolutionary evidence to identify TEs. Based on the consideration that combining structural and evolutionary information would lead to highly reliable predictions of LTR elements, LTR_INSERT was developed.

Scanning the comparative map, LTR_INSERT identified shared and specific elements. Firstly, LTR_INSERT categorized the comparative map into two partitions: alignment indels of proper size (≥ 100 bp) and well-aligned blocks. Secondly, it discovered specific elements in indel neighborhoods. If an element inserted only in one genome and the full-length structure was intact, the following signals of transposition should be observed at the target locus (Figure 1, Case A): (1) an alignment indel was composed of neither more nor less than a full-length element with a TSR at the terminal. (2) A second TSR occurred at the other terminal of the element, and this TSR had a identical counterpart in the other genome. (3) Flanking sequences of this insertion aligned well. Checking all the indels of the comparative map, LTR_INSERT selected elements that meet above criteria as specific elements. Thus, every specific elements was supported by evidences from both transposition event and structure. Thirdly, the algorithm discovered shared elements in well-aligned regions by recognizing structural characters of elements. In the second and third steps, the verification of structural characters was performed by calling LTR_FINDER, an efficient *ab initio *tool for LTR element discovery we developed recently [41]. In actual alignments, signals such as TSRs, TG-CA box and flanking strings might be placed at incorrect positions by alignment algorithm. LTR_INSERT had the capability to recognize misarranged signals.

Indels that have transposition signal (2) and (3) but lack full-length structures may be solo-LTRs originated through intra-element recombination if they further have proper sizes and TG-CA boxes. LTR_INSERT selected sequences that were similar to LTRs of full-length elements and met the above standards to be specific solo-LTRs.

We note that LTR_INSERT requires that flanking sequences of elements are well-aligned, thus to insure comparison are performed at the same locus in two genomes. In the present research, detected elements were kept only when 1 Kb flanking sequences of them shared > 90% identity between two subspecies.

With this strategy, the program predicted reliable full-length LTR retrotransposons in two genomes. The method of LTR_INSERT, i.e. "*ab initio *computation plus comparative genomics" can be directly applied to the comparison of other related genomes.

#### Identification of other LTR copies

Other LTR copies related to full-length elements were identified by searching against the two rice genomes using BLASTN [45]. When one locus matched several full-length elements, it belongs to the family of the top-hit one.

### Phylogenetic and statistical analysis

*d*_*inter *_and *d*_*intra *_values of non-coding regions were calculated by PHYLIP [46] using Kimura 2 parameters model and *d*_*s *_values of genes were calculated by PAML [47]. The multi-alignment of RT domains was constructed by CLUSTALW [48] and the phylogenetic tree was drawn by MEGA4 [49]. Statistical analyses were performed by R [50].

### Gene annotation

cDNA Verified Genes: by using the method described in [51], we selected 5548 cDNA verified *japonica *genes that have homologs in *Arabidopsis thaliana*. We searched out their counterparts in *indica *and obtained 5502 pairs at last.

BGF Predicted Gene Models: we annotated more than 30,000 gene models using BGF [51] in two rice genomes and selected 19,775 pairs of alleles with complete structures in well-aligned regions of the comparative map.

## Authors' contributions

HW carried out the design of the study, participated in LTR retrotransposon mining, data analysis and drafted the manuscript. ZX participated in developing LTR_INSERT and commented on the manuscript. HY participated in analysis of truncated LTR copies and gene model mapping between two genomes.

## Supplementary Material

Additional file 1 **The *indica-japonica *comparative map.** This file is composed of two sections: (1) The markers of the comparative map, which is the base of the following analysis and the purpose of the description is to show the high quality of the map. (2) A table gives detailed information of the *indica-japonica *comparative map. Click here for file

Additional file 2 **Dynamics of rice LTR families.** This file is a report of rice LTR families identified by LTR_INSERT. It is composed of 3 sections: (1) family information and the relationship with previously reported families. 2) Phylogenetic analysis of the 135 RT contained families. 3) Amplification pattern of rice LTR families. Click here for file

Additional file 3 **Statistical analysis of *d*_*intra *_values of elements.** This file contains 2 sections: (1) Statistical analysis supports the equality of *d*_*intra *_values in shared pairs. (2) Possible reasons for the 10% outliers of the *d*_*intra *_distributions in Figure 5. Click here for file

Additional file 4 **The distribution of *d*_*s *_of rice genes.** This file contains 2 pictures showing the *d*_*s *_distribution of rice genes. In both cases, the bimodal pattern is clear. Click here for file
