# Supplementary material for: LTR retrotransposons reveal recent extensive inter-subspecies nonreciprocal recombination in Asian cultivated rice
Source: BMC Genomics. 2008 Nov 27;9:565. doi: 10.1186/1471-2164-9-565 (PMC2612701; doi:10.1186/1471-2164-9-565)
Supplement: Additional file 1 — The indica-japonica comparative map. This file is composed of two sections: (1) The markers of the comparative map, which is the base of the following analysis and the purpose of the description is to show the high quality of the map. (2) A table gives detailed information of the indica-japonica comparative map. [file 1471-2164-9-565-S1.pdf]

Additional File 1 To: "LTR retrotransposons reveal recent extensive inter-subspecies nonreciprocal recombination in Asian cultivated rice"

Hao Wang, Zhao Xu and Hongjie Yu

The *indica-japonica* comparative map

The primary purpose of the comparative map is to construct reliable whole-genome synteny between two subspecies. We constructed the map with more than 10,000 pairs of markers. The following three aspects demonstrate their high quality.

1) Single copy number. Markers are composed of genes and long syntenic alleles (LSAs). As functional and long sequences in the genome, they are unique. As can be seen from the distribution of length (Figure S1(a)), all are longer than 1 Kb and gene markers cluster around the peak at 3 Kb. By contrast, the number of LSAs decrease slowly with the increase of size from 10 to 165 Kb, with a mean size of 26 Kb. Markers themselves cover more than one third of the genome.

2) High Density. The number of gene markers is 2.2-fold that of LSAs. The total number of LSA and gene markers is 10,462, that is, they are highly dense (3.5 Kb per marker) in the genome. Moreover, the distribution of markers is rather uniform in the genome. This can be measured by r:

$$r = \frac{length(inter\ marker\ spacer)}{length(terminal\ markers\ of\ the\ spacer)} \tag{1}$$

As shown in Figure S1(b), spacers are on average 2.2-fold longer than their terminal markers and the frequency collapses with the increase of the ratio.

3) High conservation. Both genes and LSAs share very high inter-subspecies conservation (Figure S1(c)). The mean identity reached 99.7% and the distribution of similarity obviously skews to 100%.

In summary, non-repeated, highly dense and highly similar alleles are used to build reliable synteny between two rice genomes.

These markers split two genomes into 10,474 syntenic blocks. We aligned and concatenated them to obtain 272,402 Kb matched sequences, which covered 78.3% and 73.2% of *indica* and *japonica* genomes, respectively. Details of the map are summarized in Table S1. By checking the map, for every locus in *japonica*, one can find its counterpart in *indica* and vice versa. Thus the comparative map provides a platform for allele-by-allele comparison of two genomes.

Figure S1 - Markers of the *indica-japonica* comparative map

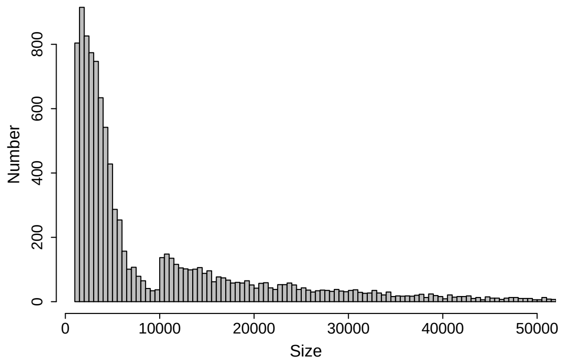

(a) Distribution of the marker size.

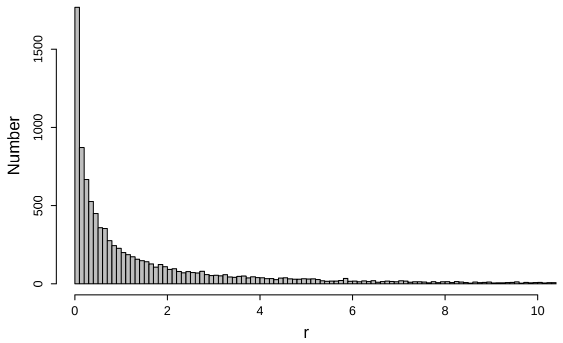

(b) Distribution of r, the ratio of inter-marker spacer to terminal markers.

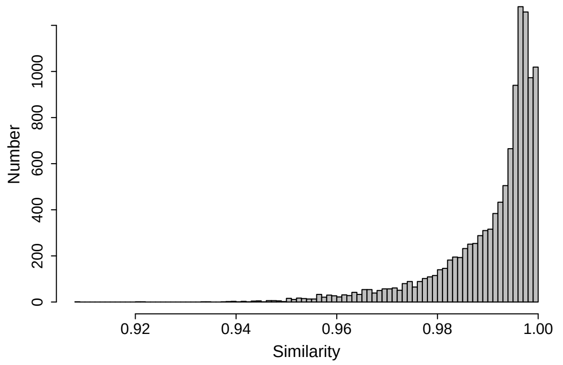

(c) Distribution of the conservation of markers

Figure S1: Three histograms reflect the high quality of the *indica* and *japonica* comparative map. a) The distribution of the marker size. Two peaks correspond to two types of markers. Gene markers are relatively short yet greater in number. Most of them are shorter than 10 Kb with a mean of 3.2 Kb. LSAs range from 6.4 to 165 Kb (only 0-50 Kb are shown here) with a mean of 26 Kb. b) The distribution of r. The number of syntenic blocks collapses with the increase of r, which means that markers are quite evenly distributed along the genome. c) Similarity of markers. All have a similarity $>$ 90% with the mean value of 99.7%.

Table S1 - Summary of the *indica*-*japonica* comparative map

Chr	Size[a](Kb)		Markers	Covered(Kb)		Coverage[b](%)		Aligned[c](Kb)	Coverage[b](%)	
	indica	japonica		indica	japonica	indica	japonica		indica	japonica
1	47,245 (43,369)	43,597(43,588)	1,658	15,894	15,905	36.6	36.5	34,641	81.7	79.5
2	38,080 (36,189)	35,925(35,920)	1,347	12,863	12,864	35.5	35.8	29,593	83.8	82.4
3	41,836 (39,028)	36,345(36,339)	1,474	16,091	16,093	41.2	44.3	31,335	82.5	86.2
4	34,661 (31,887)	35,244(35,234)	989	10,061	10,051	31.6	28.5	23,257	74.8	66.0
5	31,163 (29,054)	29,874(29,866)	990	11,450	11,457	39.4	38.4	23,351	86.5	78.2
6	32,845 (29,756)	31,247(31,209)	926	10,079	10,077	33.9	32.3	23,639	82.4	75.7
7	27,898 (26,341)	29,689(29,685)	876	8,938	8,894	33.9	30.0	21,624	84.5	72.8
8	30,367 (27,721)	28,309(28,304)	800	8,338	8,302	30.0	29.3	20,899	79.6	73.8
9	21,713 (20,778)	23,011(23,004)	661	6,891	6,842	33.2	29.7	16,983	83.2	73.8
10	22,150 (20,877)	22,877(22,863)	616	5,411	5,407	25.9	23.7	16,115	79.0	70.5
11	22,972 (21,581)	28,462(28,453)	507	4,286	4,292	19.9	15.1	14,855	72.7	52.2
12	22,948 (21,273)	27,497(27,495)	590	5,391	5,388	25.3	19.6	16,110	78.2	58.6
Total	373,878(347,854)	372,077(371,960)	11,434	115,693	115,572	33.3	31.1	272,402	78.3	73.2

[a] Numbers in brackets are sequenced sizes, that is, excluding sequencing gaps.
[b] Coverage is calculated relative to sequenced sizes, not pseudochromosome sizes.
[c] Sizes of ungapped blocks in whole-chromosome global alignments.
