# Supplementary material for: LTR retrotransposons reveal recent extensive inter-subspecies nonreciprocal recombination in Asian cultivated rice
Source: BMC Genomics. 2008 Nov 27;9:565. doi: 10.1186/1471-2164-9-565 (PMC2612701; doi:10.1186/1471-2164-9-565)
Supplement: Additional file 2 — Dynamics of rice LTR families. This file is a report of rice LTR families identified by LTR_INSERT. It is composed of 3 sections: (1) family information and the relationship with previously reported families. 2) Phylogenetic analysis of the 135 RT contained families. 3) Amplification pattern of rice LTR families. [file 1471-2164-9-565-S2.pdf]

Additional File 2 To:
"LTR retrotransposons reveal recent extensive inter-subspecies nonreciprocal recombination in Asian cultivated rice"

Hao Wang, Zhao Xu and Hongjie Yu

Contents

1 Rice LTR families identified in this study **2**

2 Phyogenetic analysis of rice LTR families **2**

3 Amplification pattern of rice LTR families **3**

3.1 LTR retrotransposons was active since the divergence of two genomes . . 3

3.2 Post-divergence insertions are balance in the two genomes 3

3.3 Observable LTR transposition events are mainly driven by about 40 predominant families 3

3.4 Pattern in the spacial dimension: whole-genome distribution 4

4 Figures and Tables **5**

4.1 Figure S2 - Phylogenetic tree of 135 LTR families that have RT domain . 5

4.2 Figure S3 - Distribution of LTR copies in *japonica* 6

4.3 Table S2: 16 novel families no hit to previously reported families 8

4.4 Table S3: 64 novel families related with previously reported families . . . 9

4.5 Table S4: 115 previously reported families 11

4.6 Table S5: Novel family candidates lacking a second copy in SET1 15

4.7 Table S6 - 43 predominant LTR retrotransposon families in rice 16

In this file, we discuss dynamics of LTR elements themselves. We report here the rice LTR families discovered in this survey, their phylogenetic relationship, their amplification patterns and the predominant families.

1 Rice LTR families identified in this study

We use SET1 and SET2 to denote all the 15916 copies identified in this study and SET2 and 3102 intergenic shared copies. Each member of SET1 has at least one unabridged LTR or covers at least 50% of a full-length element. We define LTR "family" based on sequence similarity of RT domains and LTRs. Two elements belong to the same family when identity of their RTs $\geq$ 90% or their LTRs share $\geq$ 80% identity. According to the number of family members, families are categorized into high-copy-number (≥ 100), middle-copy-number (10$\sim$100) and low-copy-number (< 10) classes.

A family is taken as novel when the following standards are met: 1) members in the family do not match entries in GenBank, Repbase, TIGR Plant Repeat Databases and RetrOryza. Here "match" means sequence similarity $\geq$ 80% with database entries; 2) two or more members of a family from different genome loci are found in SET1, at least one being full-length element. When a novel family shows homology with some previously described family but the similarity is lower than 80%, it is considered as related with that family.

By the above standards, 80 novel families were identified. All the 15916 elements were classified into 202 groups and 115 were previously described (Table S4). Similar to the situations in worm and fruit fly, though LTR retrotransposons in rice have been extensively investigated, there are novel families yet to be discovered. The present investigation discovered a total of 87 novel groups. 7 of them (Table S5) were discarded because of lack a second copy in SET1. In the 80 novel families, 64 are related with previously reported families (Table S3) while the rest 16 have no hit in the databases (BLASTN, E-Value: 10^{-10}, Table S2). It is worth noting that two of the 16 families, *Novel_63* (114 copies) and *Novel_80* (109 copies), are high-copy-number families, which means they were active in the past. In fact, half of the 16 families have more than 10 copies (Table S2).

2 Phyogenetic analysis of rice LTR families

We found RT domain in 135 families. The domains were detected by hmmsearch program in the HMMER package (`http://hmmer.janelia.org/`). The profiles of RT domain were directly downloaded from Pfam (V22.0, `pfam.sanger.ac.uk/`). The RT domain are represented by the three profiles: PF00078, PF07727 and PF05380. The result of phylogenetic analysis supports previous classification of rice LTR retrotransposons into Copia and Gypsy superfamilies. The RT phylogenetic tree is shown in Figure S2.

3 Amplification pattern of rice LTR families

3.1 LTR retrotransposons was active since the divergence of two genomes

We note that 1462, the total number of post-divergence full-length and solo- LTR insertions identified by LTR_INSERT, is an underestimation of such insertions since rather stringent criteria were applied when scanning the comparative map. Moreover, LTR_INSERT would not detect post-divergence insertions that experienced intra-genome rearrangements. Even so, the number is considerable. This indicates that LTR retrotransposons were active since after the divergence of two genomes.

3.2 Post-divergence insertions are balance in the two genomes

At first glance, the number of specific full-length elements in *japonica* is 5 times that in *indica* (601/146). The seeming imbalance gives an impression that LTR retrotransposons were far less active in *indica*. However, this "gap" can be, at least partly, explained by the masking process in the assembly of *indica* genome. In the process, reads are discarded if they contain highly repeated (more than 20 occurrences in all reads) substrings. Therefore regions tend to be absent in pseudochromosomes if they belong to middle- or high- copy-number repeats. When the masked regions overlap with or cover structure characters of an LTR element, the element will not be identified by LTR_INSERT.

Two facts support this explanation: 1) In *indica*, not a family have more than 22 full-length members while in *japonica*, some families, e.g. *hopi*, *osr25* and *retrosat1* have far more than 20 full-length copies. 2) In the specific solo-LTRs, which also reflected post-divergence insertions, the ratio dropped to 1.2, a far less significant value.

Since unmasked regions of elements can be discovered by whole-genome homologous search, we used not only full-length but also truncated elements to get more reliable estimation on the number of insertions in two subspecies. In fact, the ratio is 1.3 when investigate the difference set (SET1-SET2). Ideally, the difference set should be made of all the post-divergence insertions. However, some members in (SET1-SET2) may not come from real post-divergence transposition events but from other rearrangements. Despite this possible bias, the value is close to that in solo-LTRs and supports less difference of post-divergence insertions in the two genomes.

We therefore estimate that the activity of LTR elements were relatively balanced in two lineages.

3.3 Observable LTR transposition events are mainly driven by about 40 predominant families

We found that 80% of the post-divergence amplification was driven by only about 40 highly active families (Table S6): In the 1462 allelic specific copies, 80% (1159) belong to 43 families. They constitute 78% and 80% of all insertions in two subspecies in this period, respectively.

To see whether they were also active in the longer history of rice, we investigate all the copies in SET1. It has been shown that most observable LTR elements in rice were

inserted in less than 5 MY B.P. [1, 2]. Now SET1 cover all LTR copies identified in this work, so SET1 reflects insertions in the past 5 MY. Among the 15916 copies in SET1, there are 7293 ones from *indica* and 8623 from *japonica*. We found that the same 43 families are also highly active: these families cover 13266 members, about 83% of all SET1 copies and all of them are high-copy-number families. They are responsible for 85.1% (6063) and 83.5% (7203) insertions in *indica* and *japonica*, respectively. Further more, 20 of them have more than 100 copies in both genomes and cover 9587 (60%) copies. This result indicates that the divergence event did not significantly change the activity of these highly active families.

3.4 Pattern in the spacial dimension: whole-genome distribution

We investigated the distribution of LTR copies in the rice genome and found that they are not randomly distributed along the chromosomes. Take the distribution in *japonica* as an example (Figure S3), several trends are noticeable:

1. The LTR density is higher in the centromeric neighborhoods. In fact, the top density values occur in centromeric regions in all chromosomes except chromosome 9. Even in this exceptional chromosome, the density in the centromeric neighborhood is also high (the second-high value). This result shows that centromeric neighborhoods are LTR-dense regions.

2. The main trend of LTR density is to decrease from centromeric neighborhoods to 5' and 3' ends of chromosomes.

3. On the background of decrease, peaks and spikes disperse, e.g. 0-5 Mb of chromosome 2, 6-11 Mb of chromosome 3, 16-18 Mb of chromosome 8 and 5-8 Mb of chromosome 11. Most of them cover a region $\leq$ 5 Mb. This indicates that the insertions tend to cluster together in the scale of such size.

4. Besides centromeric neighborhoods, 5' ends of chromosomes are also LTR-dense regions. In chromosome 2, 6, 7, and 10, the density of elements recovers at the 5' ends. By contrast, density never significantly bounce back at 3' ends.

5. The LTR density are obviously imbalanced between 5' and 3' half in some chromosomes: In chromosome 4 and 9, relative to 18 Mb and 13 Mb respectively, the 5' half of chromosomes show clearly higher density than the 3' half.

Similar trends are also observed when investigating LTR copies in *indica* (data not shown).

4 Figures and Tables

4.1 Figure S2 - Phylogenetic tree of 135 LTR families that have RT domain

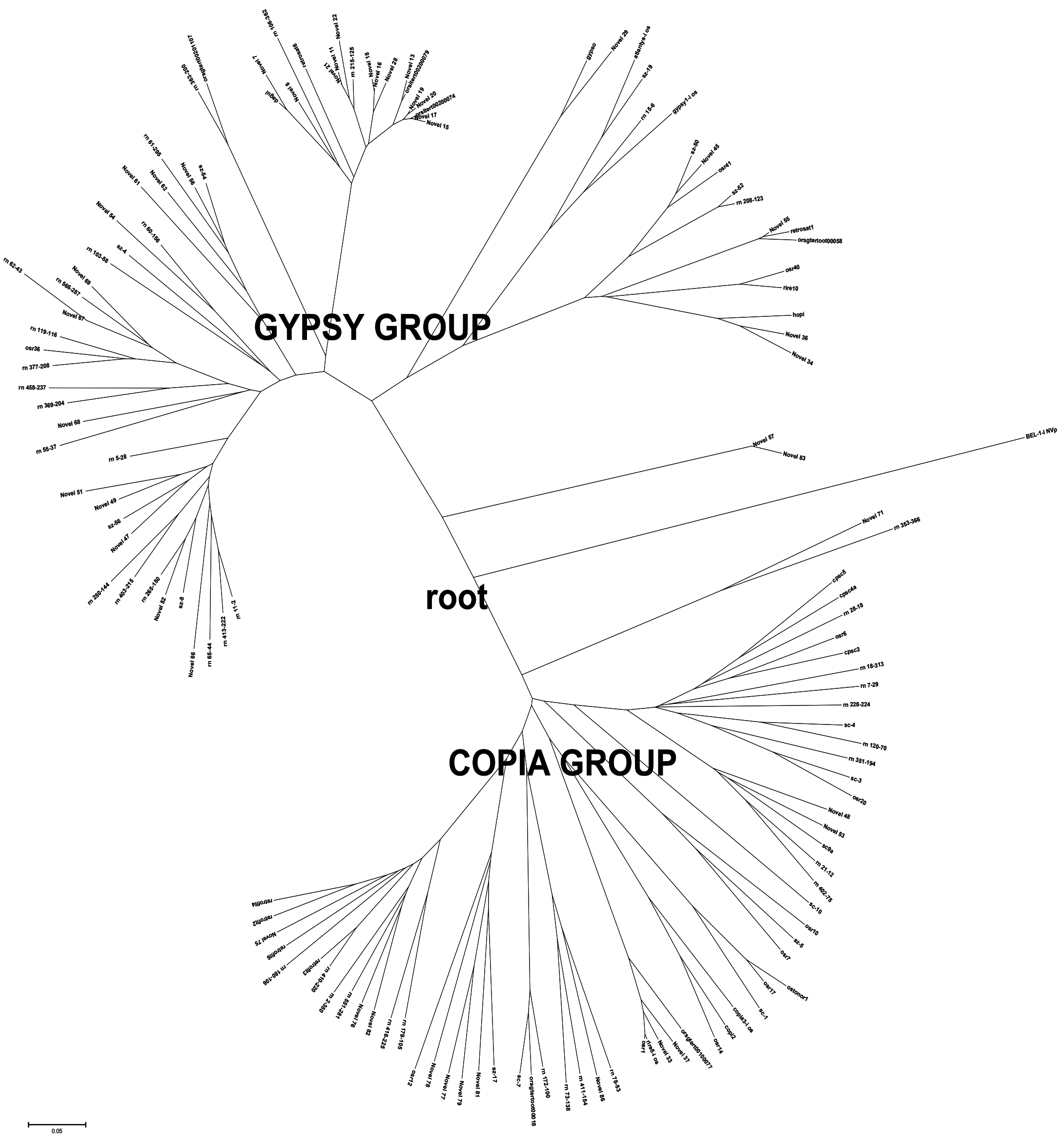

Figure S2: using the BEL-1-I_NVp (from Repbase) as outgroup, the tree is built. In this figure, RT domains of rice LTR families are clearly grouped into Copia and Gypsy superfamilies.

4.2 Figure S3 - Distribution of LTR copies in *japonica*

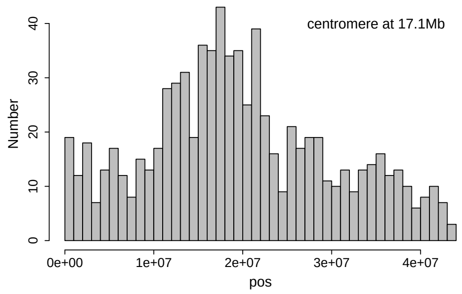

(a) chr01.

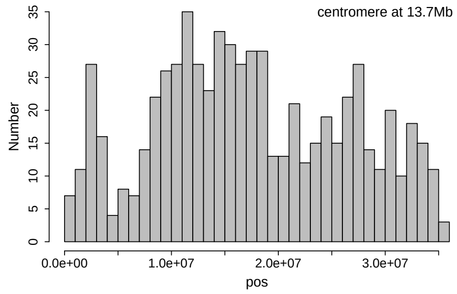

(b) chr02.

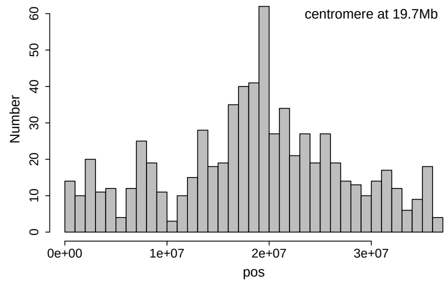

(c) chr03.

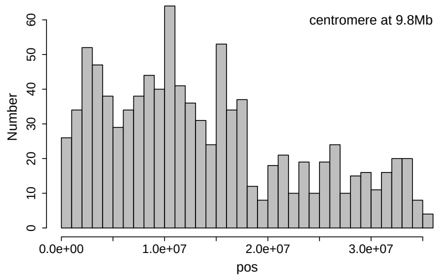

(d) chr04.

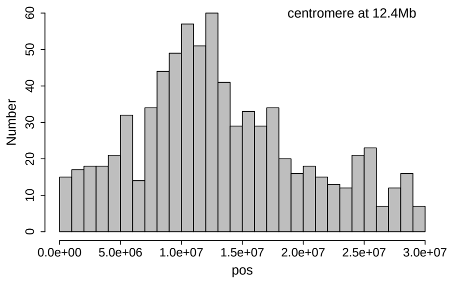

(e) chr05.

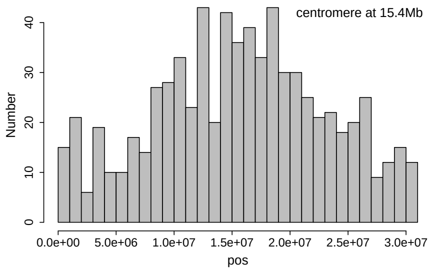

(f) chr06.

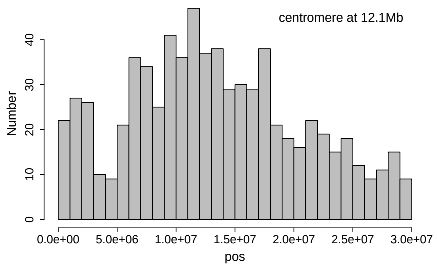

(g) chr07.

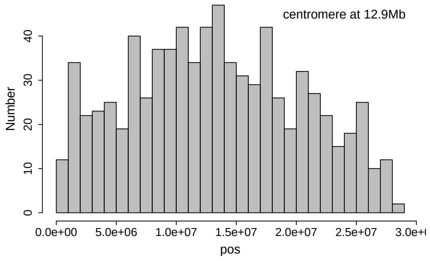

(h) chr08.

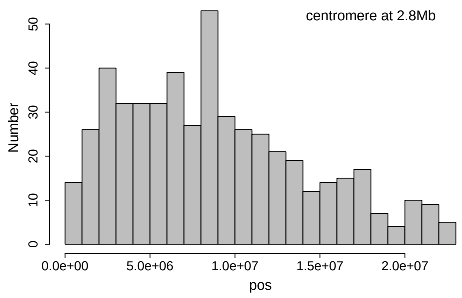

(i) chr09.

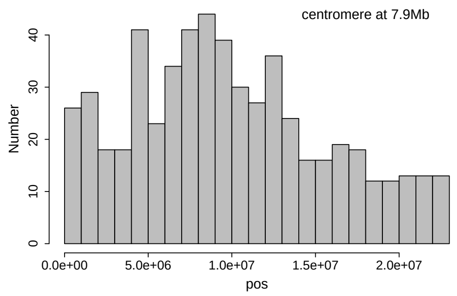

(j) chr10.

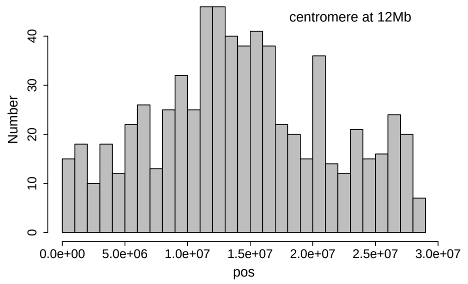

(k) chr11.

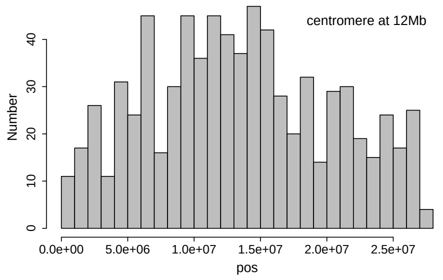

(l) chr12.

Figure S3: The distribution of 8623 LTR copies in the *japonica* genome. Locations of centromeres are shown at the upper-right of each subfigure. Each bin covers 1 Mb region.

4.3 Table S2: 16 novel families no hit to previously reported families

Sp[a]	Chr	Id	Locus[b]	LTR size	Element size	RT domain	fl[c]	all[d]
j	12	Novel_63	26323718-26326851	345:345	3134	ND	2	114
j	10	Novel_80	9013690-9016219	177:178	2530	ND	5	109
j	4	Novel_72	11434123-11435676	281:281	1554	ND	4	46
j	1	Novel_18	12437311-12449334	3104:3148	12024	3046-3555	2	34
i	1	Novel_36	7954541-7966086	915:932	11546	6434-6943	1	17
i	1	Novel_87	3011013-3012914	129:144	1902	ND	1	11
j	2	Novel_58	33547467-33550891	365:375	3425	ND	2	11
j	5	Novel_50	12758390-12760285	453:447	1896	ND	2	11
j	10	Novel_46	13130771-13132846	526:526	2076	ND	1	8
j	9	Novel_88	21768758-21770481	124:124	1724	ND	1	7
j	5	Novel_60	1387644-1390875	355:355	3232	ND	1	7
j	8	Novel_64	13915273-13918552	343:343	3280	ND	1	5
j	9	Novel_59	16622648-16625863	360:360	3216	ND	1	5
j	7	Novel_84	1080080-1084774	153:161	4695	ND	2	4
j	5	Novel_65	22893486-22897681	343:339	4196	ND	2	4
j	6	Novel_11	10213720-10268268	3248:3249	54549[e]	8582-9091	1	2

[a] The name of species. j means *japonica* and i means *indica*.
[b] Locations in the chromosomes.
[c] The number of full-length family members identified by LTR_INSERT.
[d] The number of family members in SET1.
[e] Most very long elements cover other element(s) or TEs. Here we do not eliminate these "inner" TEs.

4.4 Table S3: 64 novel families related with previously reported families

Sp	Chr	Id	Locus	LTR size	Element size	RT domain	fl	all	Related[a]
j	7	Novel_89	3398459-3400368	106:106	1910	ND	2	519	orsitert00100034
j	4	Novel_21	10937544-10949510	2973:2983	11967	3285-3794	4	330	rire8
j	8	Novel_44	15872530-15879789	572:568	7260	ND	2	318	gypsy-b
j	5	Novel_28	12713232-12734890	1488:1484	21659	8247-8756	2	219	sz-38
i	12	Novel_55	10754743-10761252	386:384	6510	1594-2001	2	203	orsgtert00201155
j	12	Novel_16	12990428-13002515	3154:3153	12088	2218-2727	8	156	rire3
j	3	Novel_45	22508954-22520943	559:579	11990	3186-3695	4	116	retrosor2
j	7	Novel_73	17811009-17812721	280:280	1713	ND	2	111	copia3-ltr_os
j	9	Novel_34	22827352-22839690	979:965	12339	2767-3240	3	105	taz
j	5	Novel_29	12797841-12807954	1268:1285	10114	5063-5614	2	97	orsitertoot00060
j	11	Novel_74	20867255-20869012	257:257	1758	ND	3	94	orsiotot00000037
i	10	Novel_24	12068758-12077892	2832:2836	9135	ND	2	89	rn_215-125
j	9	Novel_22	5523501-5535431	2926:2969	11931	3297-3806	2	72	orsitert00200152
i	12	Novel_33	7215555-7222438	1085:1120	6884	2649-3422	1	67	orsitert00100074
j	2	Novel_41	16838684-16843743	665:668	5060	ND	2	56	bajiein
j	5	Novel_42	13366572-13377943	586:586	11372	ND	2	55	orsitertoot00269
i	10	Novel_26	16424540-16432376	2793:2833	7837	ND	1	54	rn_215-125
j	10	Novel_15	10031939-10044516	3160:3128	12578	2217-2726	1	41	osr34
j	8	Novel_31	18506098-18512450	1189:1224	6353	ND	2	35	sz-30
i	7	Novel_25	6864012-6872532	2822:2833	8521	ND	1	32	rn_215-125
j	2	Novel_4	13773435-13785384	4174:4173	11950	ND	4	32	rire3a
j	12	Novel_39	26538753-26543045	706:639	4293	ND	2	29	bajiein
j	6	Novel_17	8199155-8226565	3137:3137	27411	3530-4039	1	24	retrosat1
j	1	Novel_19	34177192-34188747	3103:3101	11556	2218-2727	1	23	retrosat2_i
j	6	Novel_20	12477444-12488894	3039:3056	11451	2630-3139	1	22	retrosat2_i
j	10	Novel_71	9211961-9219641	293:283	7681	1055-1756	1	17	orsitertoot00419
j	6	Novel_38	14847766-14852112	745:782	4347	ND	2	16	orsitertoot00109
j	7	Novel_8	8633233-8646919	3729:3714	13687	2217-2726	1	16	orsitert00200145
i	4	Novel_23	7296254-7303810	2904:2867	7557	ND	2	14	truncator2_os
j	7	Novel_2	20660597-20672347	4251:4248	11751	ND	1	14	rire3a
j	11	Novel_7	19698617-19736982	3860:3868	38366	2216-2725	1	11	retrosat4
j	6	Novel_77	24823456-24828265	204:204	4810	733-1503	1	10	rn_66-340
j	2	Novel_5	14794240-14805744	4053:4107	11505	ND	1	10	rire3a
i	8	Novel_78	24480974-24485696	189:189	4723	725-1495	1	9	zrsstertoot06832

continued...

Sp	Chr	Id	Locus	LTR size	Element size	RT domain	fl	all	Related
j	5	Novel_13	2664848-2676599	3173:3196	11752	2218-2727	1	9	retrosat2ltra
i	2	Novel_57	31861844-31866929	378:378	5086	1964-2233	1	9	orsitertoot00427
j	11	Novel_68	18387188-18392658	320:320	5471	2130-2639	2	8	orsitertoot00362
i	9	Novel_79	14289744-14294045	167:167	4302	726-1496	3	6	zrsstertoot45619
j	9	Novel_3	10500982-10512297	4208:4210	11316	ND	1	6	rire3_p0698g03-1
j	7	Novel_35	25415634-25420755	955:955	5122	ND	1	6	orsitetnoot00104
j	2	Novel_62	24391216-24397613	348:348	6398	3098-3607	1	5	sz-21ltr
j	3	Novel_83	11582786-11587224	167:167	4439	1112-1453	2	5	orsstert00100092
j	1	Novel_86	31449160-31454175	134:134	5016	3266-4027	3	5	hrsitert00100178
j	11	Novel_1	2028550-2039854	4618:4629	11305	ND	1	4	sz-48ltr
j	6	Novel_40	3715283-3719077	697:692	3795	ND	2	4	sz-29ltr
i	8	Novel_48	20652822-20658140	481:480	5319	736-1506	1	4	rn_21-12
j	11	Novel_66	14894464-14900063	343:341	5600	2299-2808	2	4	osr38_i
j	4	Novel_54	33788576-33794600	413:413	6025	2120-2503	1	4	orsitertoot00384
j	7	Novel_37	6991728-6997280	839:844	5553	1831-2604	2	4	orsitert00100074
j	7	Novel_10	12402334-12411238	3358:3358	8905	ND	3	4	gypsy-a
i	8	Novel_49	2841490-2852722	460:460	11233	7667-8176	1	3	orsitertoot00442
j	6	Novel_61	23164430-23169540	350:350	5111	2097-2606	2	3	orsitertoot00427
i	5	Novel_75	24813760-24818608	228:216	4849	726-1496	1	3	orsitertoot00412
j	1	Novel_14	8638729-8651529	3164:3165	12801	ND	1	3	gypsy-a
j	8	Novel_51	1297044-1302701	445:445	5658	2118-2627	2	2	sz-8
i	10	Novel_81	6322686-6327977	173:167	5292	727-1497	1	2	sz-57
j	3	Novel_56	2598193-2603421	388:388	5229	1845-2354	2	2	sz-54
i	9	Novel_53	6262176-6267339	415:415	5164	2829-3599	1	2	rn_21-12
j	1	Novel_27	13607236-13617494	2637:2637	10259	ND	1	2	rire3_p0698g03-1
j	2	Novel_32	24247296-24252844	1170:1172	5549	ND	2	2	orsitetnoot00104
j	7	Novel_69	5200351-5205804	318:318	5454	2137-2646	2	2	orsitertoot00364
i	9	Novel_67	13887112-13892539	335:335	5428	2396-2650	1	2	orsitertoot00354
j	3	Novel_30	8949177-8954989	1245:1245	5813	ND	1	2	orsgtertoot00073
j	10	Novel_9	13236070-13244833	3553:3556	8764	ND	1	2	mesaaw

[a] The best matched LTR elements in GenBank, TIGR, Repbase and RetrOryza.

4.5 Table S4: 115 previously reported families

Sp	Chr	Name[a]	Locus	LTR size	Element size	RT domain	fl	all
j	1	osr25	8232600-8239440	417:417	6841	ND	102	1110
j	1	hopi	29671117-29684032	1088:1089	12916	2742-3251	189	1083
j	9	bajieltr	9587447-9591855	711:708	4409	ND	14	948
j	2	sz-5	24411276-24420590	1224:1225	9315	1819-2589	4	700
j	8	rire10	19728961-19740440	564:562	11480	6492-7001	12	576
j	10	retrosat1	21163707-21175008	441:441	11302	3227-3736	73	535
j	6	orsgtert00100077	23042219-23048651	960:972	6433	2461-3231	49	533
j	6	orsitertoot00029	22421802-22422514	280:242	713	ND	2	394
j	6	sz-19	10761641-10775443	1496:1505	13803	6185-6664	4	370
j	9	gypsy1-i_os	10293694-10302582	654:654	8889	4322-4840	6	350
j	1	sz-58	6404797-6405204	115:115	408	ND	2	328
j	7	orsgtertoot00058	16232389-16246625	440:439	14237	6686-7195	3	326
i	6	orsitertoot00175	15780029-15785757	794:794	5729	ND	18	266
j	10	osr10	16542752-16554661	1527:1571	11910	4296-5066	1	238
j	2	wombat	30499592-30502952	397:397	3361	ND	4	233
j	4	dagul	31722831-31736825	3903:3911	13995	3457-3966	14	233
j	12	sc-3	24733624-24739377	392:392	5754	744-1514	35	213
j	1	osr41	37755916-37767027	514:532	11112	6372-6881	9	211
j	1	orsitertoot00221	21244693-21249670	788:787	4978	ND	6	211
j	5	orsitertoot00028	14891822-14900406	3392:3423	8585	ND	5	209
j	8	orsgotot00000180	9665344-9666274	202:202	931	ND	2	193
j	2	rire5-i_os	10621622-10628085	1020:976	6464	2440-3213	10	187
j	8	copia3-i_os	25590512-25595591	262:262	5080	822-1601	2	186
j	8	rire3a	15001482-15011995	3179:3179	10514	ND	16	182
j	1	ostonor1	25893706-25900160	492:492	6455	900-1676	15	164
i	2	orsgtert00200863	15314492-15322228	3001:3002	7737	ND	16	164
j	7	osr40	6159057-6170438	564:564	11382	6436-6945	5	163
j	10	osr1	18291895-18298293	965:965	6399	1257-2030	11	156
j	4	gypso	4220119-4229878	1200:1191	9760	2111-2380	2	146
j	11	osr17	6144831-6150820	501:501	5990	3318-3518	18	124
j	5	rn_215-125	14202857-14214632	2862:2861	11776	2218-2727	2	120
j	10	orsitert00200074	4529957-4541635	2713:2704	11679	3536-4045	3	114
j	7	osr14	8224245-8228901	319:319	4657	773-1453	2	97
j	1	szltr	25069705-25075282	1268:1262	5578	ND	2	88

continued...

Sp	Chr	Name	Locus	LTR size	Element size	RT domain	fl	all
j	9	rcb11	3085280-3089692	793:793	4413	ND	5	87
j	8	rn_363-200	13327558-13335312	908:908	7755	3515-4024	4	79
j	9	orsitert00200079	13101533-13113112	3094:3093	11580	2667-3176	8	77
j	1	sz-22	9953399-9957802	769:768	4404	ND	7	68
j	1	atlantys-i_os	30116633-30127802	127:127	11170	4123-4638	2	62
j	10	orsgtert00201107	9953028-9960642	858:858	7615	3474-3983	1	58
j	5	orsitertoot00114	24138438-24142894	781:792	4457	ND	1	57
i	5	sc-1	21588069-21594988	400:400	6920	4250-5026	2	45
i	10	sz-50	2758923-2770559	504:504	11637	3201-3710	1	41
j	12	copi2	21072041-21076753	283:283	4713	2549-3328	3	40
j	2	orsgtertoot00018	33941048-33946248	146:146	5201	3415-4185	7	37
j	2	orsitertoot00160	18879183-18883603	791:789	4421	ND	2	35
j	11	taz	19550959-19560628	986:930	9670	ND	2	31
j	3	sc-10	15013016-15017934	361:361	4919	2705-3475	2	29
j	9	osr7	17434195-17444526	1636:1629	10332	2021-2791	5	25
j	2	sc9a	2527396-2532695	478:478	5300	736-1506	2	20
j	4	rn_78-53	16500479-16503431	143:139	2953	1182-1946	1	20
j	8	rn_351-194	1841744-1846731	287:287	4988	2901-3671	2	18
j	9	rn_172-100	11857141-11861865	122:122	4725	725-1495	2	17
j	8	osr44	24407680-24408886	157:156	1207	ND	4	17
j	1	osr36	5732822-5737976	319:317	5155	2121-2630	4	17
j	9	rn_353-366	13251480-13258264	269:269	6785	4381-5163	2	16
j	6	sz-52	28599824-28612915	746:746	13092	7882-8391	4	15
j	7	rn_314-187	7768908-7773073	794:793	4166	ND	2	13
j	7	sc-7	5612835-5617264	117:117	4430	2704-3474	4	12
j	4	rn_208-123	26865036-26877997	738:736	12962	3108-3617	2	12
j	6	rn_179-105	7468720-7473519	208:208	4800	2885-3658	3	12
j	12	rn_61-295	14208938-14213961	342:342	5024	1850-2245	2	11
j	6	retrofit3	7387596-7392660	325:325	5065	2920-3690	4	11
j	1	osr12	39729892-39734606	219:219	4715	727-1497	3	11
j	1	rn_7-29	18213920-18219127	436:436	5208	739-1509	2	10
j	5	retrofit4	26123537-26128345	204:204	4809	2906-3676	2	10
j	1	rn_18-313	41179919-41185035	360:360	5117	2882-3652	1	9
j	1	mesaaw	29580208-29588952	3564:3564	8745	ND	1	9
i	9	cpsc5	7765972-7771092	406:406	5121	735-1487	1	9

continued...

Sp	Chr	Name	Locus	LTR size	Element size	RT domain	fl	all
j	4	sc-4	14082740-14087958	421:421	5219	2872-3642	1	8
j	5	rn_119-116	17976084-17981418	302:302	5335	2292-2624	2	8
j	2	osr20	22951582-22957120	284:284	5539	3487-4203	2	8
j	5	cpsc4a	21490550-21495734	408:408	5185	2864-3634	5	8
j	9	rn_413-222	15973999-15979458	341:341	5460	2105-2614	2	7
j	6	rn_411-154	22803893-22809034	125:125	5142	746-1510	2	7
j	1	osr6	8819344-8824546	439:439	5203	917-1504	2	7
j	5	rn_73-138	22862627-22868262	148:148	5636	725-1489	3	6
j	2	rn_55-37	9914453-9919999	365:365	5547	2146-2517	2	6
j	6	rn_250-144	27769708-27775334	423:423	5627	2155-2664	2	6
j	2	rn_15-6	15239910-15249199	822:826	9290	2610-3128	2	6
i	1	rn_103-58	24718174-24724314	441:441	6141	2118-2627	1	6
j	7	retrofit6	23484198-23489000	205:205	4803	2905-3669	1	6
j	4	orsitertoot00164	11108619-11111269	793:775	2651	ND	2	6
j	4	cpsc3	17268523-17273603	429:428	5081	2722-3234	2	6
i	8	rn_410-220	19754458-19763884	278:281	9427	7373-8143	1	5
j	9	rn_228-224	9825875-9831006	378:378	5132	2868-3638	2	5
j	2	rn_62-43	18891071-18896465	318:340	5395	2337-2654	2	4
j	1	rn_5-28	35297314-35302973	454:455	5660	2124-2354	2	4
j	3	rn_402-75	32140284-32145951	473:472	5668	3440-3988	4	4
i	12	rn_377-208	10842677-10848026	299:300	5350	2122-2631	2	4
j	1	rn_28-18	28664569-28669753	423:423	5185	2834-3604	2	4
j	8	rn_2-350	25810775-25815566	173:173	4792	726-1496	3	4
j	1	rn_21-12	30208178-30213452	461:462	5275	736-1506	3	4
j	4	rn_180-106	16672320-16686345	216:217	14026	3141-3797	2	4
j	3	rn_120-70	17516342-17521466	413:407	5125	703-1353	2	4
j	9	retrosat6	5042059-5056998	4235:4236	14940	2215-2724	1	4
j	11	gypsy-a	5714401-5723350	3367:3367	8950	ND	1	4
i	9	sz-17	7958915-7964100	135:135	5186	724-1494	1	3
i	12	rn_551-281	16404215-16409031	200:200	4817	726-1496	2	3
j	10	sz-8	22788271-22793768	349:352	5498	2278-2604	2	2
j	7	sz-56	28661147-28666795	425:425	5649	2115-2624	2	2
j	6	sz-40	25840852-25849627	3405:3405	8776	ND	1	2
j	10	sz-4	14395729-14401730	427:439	6002	2502-3011	2	2
j	2	rn_65-44	26676698-26682101	330:330	5404	2124-2630	2	2

continued...

Sp	Chr	Name	Locus	LTR size	Element size	RT domain	fl	all
j	10	rn_458-237	12175138-12180483	344:346	5346	2022-2531	2	2
j	9	rn_416-225	18058371-18063171	232:229	4801	2845-3615	2	2
j	9	rn_403-215	4418945-4424595	422:422	5651	2118-2627	1	2
j	8	rn_369-204	15467340-15472579	284:281	5240	2130-2639	2	2
j	1	rn_11-2	7746007-7751460	332:333	5454	2115-2624	2	2
j	3	retrofit2	17538028-17542801	209:209	4774	2860-3630	1	2
j	7	sz-54	4240914-4246115	391:391	5202	1806-2315	1	1
j	6	rn_60-156	17058825-17064798	424:424	5974	2115-2624	1	1
j	12	rn_566-287	13663516-13668988	324:324	5473	2154-2663	1	1
j	6	rn_265-150	8825950-8831588	436:436	5639	2115-2624	1	1
j	9	rn_106-362	5195813-5207691	3118:3118	11879	2922-3428	1	1

[a] Family ID in GenBank, TIGR, Repbase and RetrOryza.

4.6 Table S5: Novel family candidates lacking a second copy in SET1

Sp	Chr	Id	Locus	LTR size	Element size	RT domain	fl	all	Related
i	9	Novel_52	19967658-19973275	429:438	5618	2109-2618	1	1	rn_265-150
i	10	Novel_70	10697648-10701532	297:297	3885	ND	1	1	orsitertoot00436
i	7	Novel_47	22301410-22307183	492:492	5774	2118-2627	1	1	orsitertoot00368
i	11	Novel_82	3921617-3926404	171:171	4788	726-1496	1	1	orsitertoot00320
j	8	Novel_76	11018912-11023780	209:209	4869	2956-3726	1	1	orsitertoot00320
j	12	Novel_12	2492262-2500369	3226:3226	8108	ND	1	1	gypsy-a
j	10	Novel_43	16557649-16561266	584:584	3618	ND	1	1	ND

4.7 Table S6 - 43 predominant LTR retrotransposon families in rice

Family	Specific (FL+Solo)		Shared	All copies		Total
	indica	japonica	(FL+TC)[a]	indica	japonica	
osr25[b]	28	82	138	461	649	1110
hopi	127	268	51	555	528	1083
bajieltr	1	7	282	471	477	948
sz-5	3	2	143	339	361	700
rire10	0	2	142	281	295	576
retrosat1	25	49	63	281	254	535
orsgtert00100077	10	58	73	241	292	533
Novel_89[c]	0	0	175	249	270	519
orsitertoot00029	3	4	64	193	201	394
sz-19	5	2	98	162	208	370
gypsy1-i_os	2	1	77	177	173	350
Novel_21	3	6	19	118	212	330
sz-58	0	0	115	167	161	328
orsgtertoot00058	0	1	82	157	169	326
Novel_44	0	0	83	147	171	318
orsgtertoot00099	22	12	58	155	111	266
osr10	0	1	58	117	121	238
wombat	0	0	87	114	119	233
dagul	12	72	5	35	198	233
Novel_28	1	3	46	104	115	219
sc-3	12	17	40	116	97	213
osr41	0	1	40	98	113	211
orsitertoot00221	1	2	70	110	101	211
orsitertoot00028	14	24	18	67	142	209
Novel_55	2	0	65	106	97	203
orsgotot00000180	1	0	67	99	94	193
rire5-i_os	4	9	21	86	101	187
copia3-i_os	0	0	50	90	96	186
rire3a	1	38	0	7	175	182
ostonor1	1	14	11	39	125	164
orsgtert00200863	46	49	11	62	102	164
osr40	1	2	42	77	86	163
osr1	4	17	34	70	86	156
Novel_16	3	27	8	19	137	156
gypso	0	0	37	77	69	146
osr17	5	15	13	45	79	124
rn_215-125	27	3	3	80	40	120
Novel_45	0	0	33	58	58	116
orsitert00200074	1	3	3	25	89	114
Novel_63	0	0	43	53	61	114
Novel_73	0	0	39	58	53	111
Novel_80	0	2	33	47	62	109
Novel_34	1	0	37	50	55	105
Sum	366	793	2577	6063	7203	13266

[a] FL and TC are the abbreviations of full-length and truncated element, respectively.
[b] Families sharing ≤ 80% sequencing similarity with known elements are named by their names in one of the 4 LTR element databases: GenBank, Repabse, TIGR Plant Repeats Databases and RetrOryza ([see Additional file 2] for details

[c] Novel families discovered in this study are named as "Novel_X", where "X" is an integer.

References

[1] J. Ma and J. L. Bennetzen. Rapid recent growth and divergence of rice nuclear genomes. *Proc Natl Acad Sci U S A*, 101(34):12404–10, 2004.

[2] C. Vitte, O. Panaud, and H. Quesneville. LTR retrotransposons in rice (Oryza sativa, L.): recent burst amplifications followed by rapid DNA loss. *BMC Genomics*, 8:218, 2007.
