# Supplementary material for: LTR retrotransposons reveal recent extensive inter-subspecies nonreciprocal recombination in Asian cultivated rice
Source: BMC Genomics. 2008 Nov 27;9:565. doi: 10.1186/1471-2164-9-565 (PMC2612701; doi:10.1186/1471-2164-9-565)
Supplement: Additional file 3 — Statistical analysis of dintra values of elements. This file contains 2 sections: (1) Statistical analysis supports the equality of dintra values in shared pairs. (2) Possible reasons for the 10% outliers of the dintra distributions in Figure 5. [file 1471-2164-9-565-S3.pdf]

Additional File 3 To: "LTR retrotransposons reveal recent extensive inter-subspecies nonreciprocal recombination in Asian cultivated rice"

Hao Wang, Zhao Xu and Hongjie Yu

Contents

1 Statistical analysis supports the equality of d_{intra} values in shared pairs 1

2 Possible reasons for the 10% outliers in Figure 5 1

1 Statistical analysis supports the equality of d_{intra} values in shared pairs

We have shown that Group-A elements are orthologous pairs, thus they diverged with the genome divergence. Therefore, one expects that the difference of d_{intra} values of the 66 Group-A pairs obey a normal distribution. In fact, they do pass normality tests (Shapiro-wick test P-Value: 0.30 and Kolmogorov-Smirnov test P-Value: 0.44). Hence, it is highly possible that their d_{intra} values are statistically equal.

Regarding the 42 Group-B elements, because most of them experienced ISNR at different time, the d_{intra} differences of them do not obey normality distribution anymore. However, since the d_{intra} differences of each pair accumulated after ISNR, we expect that many of them show $d_{intra}\ difference \leq d_{inter}$. In fact, this inequality holds in more than 80% pairs and 26 pairs (61%) have identical d_{intra} values in two subspecies. Therefore, the d_{intra} difference of Group-B elements are not significant.

Overall, d_{intra} values of each pairs are equal in two subspecies. Hence it is proper to use their average to represent d_{intra} of each pair.

2 Possible reasons for the 10% outliers in Figure 5

In Figure 5, we inferred that the two rice genomes diverged at D and $D \in (0.014,\ 0.017)$ based on the separation of d_{intra} values of specific and shared elements. 90% elements show expected d_{intra} values, i.e. the values in share elements are greater than D and in specific elements are less than D. It is worth noting that 90% is a strong statistical support to our statement. Even so, here we try to explain the 10% outliers.

Although we now lack enough information to find the exact reason for each abnormal d_{intra} value in specific elements, we can explain some unusual d_{intra} values of shared pairs. For example, we have explained in the section "Many LTR retrotransposon-contained loci have been converted by ISNR" that q_1 and q_2 are very likely inserted posterior to the genome divergence If so, it is natural that they have small d_{intra} values.

Overall, there are many reasons may lead to abnormal substitution number. We list three important below: 1) substitutions are always affected by random events and this may make some elements show d_{intra} values unexpectedly large or small; 2) higher evolutionary speed in some loci and 3) sequencing errors.
