# Supplementary material for: LTR retrotransposons reveal recent extensive inter-subspecies nonreciprocal recombination in Asian cultivated rice
Source: BMC Genomics. 2008 Nov 27;9:565. doi: 10.1186/1471-2164-9-565 (PMC2612701; doi:10.1186/1471-2164-9-565)
Supplement: Additional file 4 — The distribution of ds of rice genes. This file contains 2 pictures showing the ds distribution of rice genes. In both cases, the bimodal pattern is clear. [file 1471-2164-9-565-S4.pdf]

Additional File 4 To: "LTR retrotransposons reveal recent extensive inter-subspecies nonreciprocal recombination in Asian cultivated rice"

Hao Wang, Zhao Xu and Hongjie Yu

Figure S4 - The distribution of d_s of rice genes

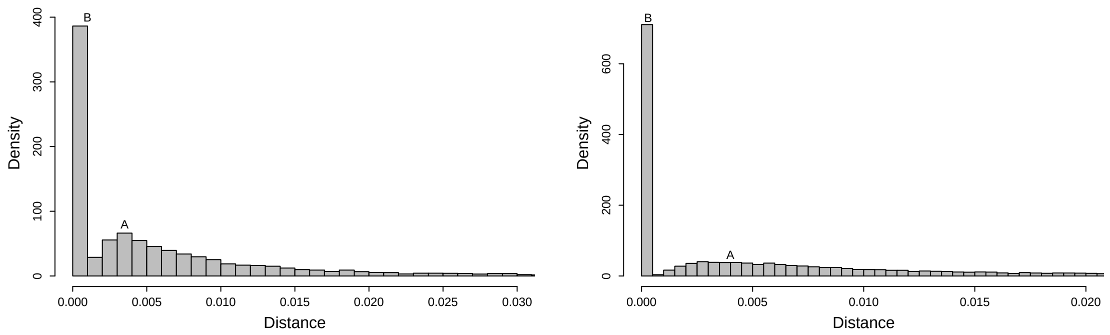

(a) d_s of allelic cDNA verified genes.

(b) d_s of allelic BGF predicted genes.

Figure S4: d_s of rice genes. Bimodal pattern is clear in both data sets. The proportion of Group-B genes is 27.3% and 25.6% in cDNA verified genes and BGF annotated genes, respectively.
